# Military, Medical and Surgical Essays—Prepared for the United States Sanitary Commission

**Published:** 1864-10

**Authors:** 


### BOOKS REVIEWED.

*Military, Medical and Surgical Essays—Prepared for the United States Sanitary Commission—Edited by* William A. Hammond, M. D., *Surgeon-General U. S. Army, etc. Philadelphia:* J. B. Lippincott & Co., 1864.

This work consists of a collection of essays on different medical and surgical subjects, which were prepared at the request of the Sanitary Commission, and published in pamphlet form for gratuitous circulation among the medical officers of the army. They have been collected and arranged by Dr. Hammond under three general heads, viz: those relating to the prevention of disease, those relating to medical subjects, and those relating to surgical subjects.

These essays are all valuable, and present the latest published views on the subjects of which they treat, so that they are valuable for surgeons in the field who are debarred from carrying more voluminous works. The papers are all well written and valuable as a whole, and we shall be perhaps pardoned for suggesting that those on medical and surgical subjects are of more value than those on hygienics, not because hygiene is not worth more in an army than therapeutics, but because the suggestions of civilians in regard to the former, though true, are not so likely to be practicable, while the general truths of medicine and surgery are equally as true in the field as at home. These papers on hygiene may be commended to those young men who are just entering upon a career in the army medical staff, and we are sure their suggestions will be valuable, though not always practicable in an active campaign. We have always held the opinion indeed, that the field and line officers need more instruction on these subjects than the medical officers, for they must carry out the regulations which the surgeon adopts. We regret that we have not time to notice these papers *seriatim*, but must content ourselves with general commendation of their worth.

The papers of the second class, on medical subjects are all valuable, both as well prepared monographs, and as timely memoranda for the surgeon, cut off from all communion with bulkier tomes. It would be invidious to attempt to draw any distinctions where all are so excellent, and we must content ourselves with a cursory notice of each. The first is from the pen of Dr. Hammond himself, on the subject of scurvy, and is the best article in regard to that disease which we have seen. It effectually explodes the crude ideas and false theories in regard to that disease which were, and perhaps still are afloat in the profession. The notion has been universally adopted that a diet of salted meat was the great cause of scurvy, and much unnecessary sympathy has been expended on our soldiers beeause they must eat such food. But Dr. Hammond has shown conclusively, what army surgeons have everywhere observed, that there is no one article of food which can cause this disease. We have ourselves seen men have scurvy when they had plenty of both salt and fresh meat, and when they had enough of fresh but no salt food. The causes of this disease are not only to be looked for in the diet list, but also in all the physical and moral conditions which pertain to the lot of the overworked, and depressed soldier or sailor. The treatment must of course correspond. The diet must be regulated, so as to give the men not only fresh vegetables, but a pleasing variety of food, and all severe labor and depressing circumstances must be carefully removed. Medicines are but little called for in these cases, and are only applicable as adjuvants in removing special complications. The author recommends the use of the muriated tincture of iron as a remedy for the anæmia which is always present, and we can bear testimony to its usefulness.

The next paper, on miasmatic fevers, by Dr. John T. Metcalfe, is a concise and well-written *resume* of the pathology and treatment of this most troublesome class of diseases in our Southern States. We would call particular attention to one sentence, which has been very appropriately italicised, and which we wish every physician who is called on to treat these fevers, could "read, mark, and inwardly digest;" it is as follows: "*It may be taken as an axiom, that the sooner we produce the state of cinchonism, the more speedily and certainly the disease will be subdued.*"

The next two papers, on continued fevers, by J. Baxter Upham, M. D., and on yellow fever, by Dr. Metcalfe, seem to be, from the cursory examination we have been able to give them, well digested synopsis of the latest views of the profession on these important subjects.

The fact that the next paper, on pneumonia, was written by our former townsman, Prof. Austin Flint, precludes the necessity of our saying any thing about it to the profession in Western New York.

The last essay on medical subjects, on dysentery, by Alfred Stille, M. D., is a very full, carefully prepared, and consequently valuable monograph on that great scourge of all armies, and will repay a careful perusal.

Of the surgical essays three seem to us particularly valuable, viz: one on amputations, by Dr. Stephen Smith; on the excision of joints for traumatic cause, by Dr. R. M. Hodges, and the closing one on venereal diseases, by Dr. Bumstead.

In closing this imperfect and hastily prepared notice of this book, we would cordially commend it to all young men especially, who are about entering the medical staff, believing they will find in it many valuable suggestions and timely hints. The high character of all the authors concerned in its production is a guarantee of the general correctness and value of all they have said. Neither can we avoid the reflection that many physicians in civil life would find these brief memoranda useful on many occasions. P.

